# Case report: malignant hypertension associated with catecholamine excess in a patient with Leigh syndrome

**DOI:** 10.1186/s40885-022-00231-4

**Published:** 2023-03-01

**Authors:** Ana Solis, Joshua Shimony, Marwan Shinawi, Kevin T. Barton

**Affiliations:** 1 Department of Pediatrics, Washington University in St Louis School of Medicine, 660 S. Euclid Ave, St Louis, MO 63110 USA; 2 Mallinkrodt Institute of Radiology, Washington University in St Louis School of Medicine, 4525 Scott Avenue Campus, Box 8131, St Louis, MO 63141 USA

**Keywords:** Pediatrics, Nephrology, Leigh syndrome, Hypertension, Secondary hypertension

## Abstract

**Background:**

Leigh syndrome is a progressive neurodegenerative mitochondrial disorder caused by multiple genetic etiologies with multisystemic involvement that mostly affecting the central nervous system with high rate of premature mortality.

**Case presentation:**

We present a 3-year, 10 month-old female patient with Leigh syndrome complicated by renal tubular acidosis, hypertension, gross motor delay, who presented with hypertensive emergency, persistent tachycardia, insomnia and irritability. Her previous genetic workup revealed a pathogenic variant in the *MT-ND5* gene designated as m.13513G > A;p.Asp393Asn with a heteroplasmy of 69%. She presented acutely with malignant hypertension requiring intensive care unit admission. Her acute evaluation revealed elevated serum and urine catecholamines, without an identifiable catecholamine-secreting tumor. After extensive evaluation for secondary causes, she was ultimately found to have progression of her disease with new infarctions in her medulla, pons, and basal ganglia as the most likely etiology of her hypertension. She was discharged home with clonidine, amlodipine and atenolol for hypertension management. This report highlights the need to recognize possible autonomic dysfunction in mitochondrial disease and illustrates the challenges for accurate and prompt diagnosis and subsequent management of the associated manifestations. This association between catecholamine induced autonomic dysfunction and Leigh syndrome has been previously reported only once with *MT-ND5* mutation.

**Conclusions:**

Elevated catecholamines with malignant secondary hypertension may be unique to this specific mutation or may be a previously unrecognized feature of Leigh syndrome and other mitochondrial complex I deficient syndromes. As such, patients with Leigh syndrome who present with malignant hypertension should be treated without the need for extensive work-up for catecholamine-secreting tumors.

## Background

Leigh syndrome (subacute necrotizing encephalomyelopathy) (MIM# 256000) describes a progressive neurodegenerative mitochondrial disorder with heterogenous genetic etiology. All underlying genetic abnormalities lead to mitochondrial dysfunction via alteration of the oxidative phosphorylation in the mitochondrial respiratory chain complexes and ultimately result in ATP deficiency, accumulation of lactate in body fluids, and other metabolic derangements. Leigh Syndrome is the prototypical mitochondrial syndrome presenting with dysfunction in myriad organ systems but mostly affecting the central nervous system as evidenced by the typical brain MRI findings of focal and bilateral lesions in the basal ganglia, brain stem, cerebellum, and white matter changes. The most common neurologic manifestations include hypotonia, developmental delay/intellectual disability, epilepsy, ataxia, movement disorder, and poor oromotor coordination [1]. Additional symptoms can include poor feeding, cardiomyopathy, cardiac arrhythmias, and respiratory failure [2]. Autonomic dysfunction has been rarely reported with mitochondrial disease in the form of tachycardia, hyperhidrosis, diarrhea and vomiting [3].

We describe a patient with Leigh syndrome caused by a pathogenic variant in the *MT-ND5* gene (MIM# 516005) designated as m.13513G > A;p.Asp393Asn with a heteroplasmy of 69%. Her clinical course was complicated by hypertensive emergency, persistent tachycardia, and elevated catecholamines. In this report, we discuss the clinical presentation and pathophysiology of this rare complication and our management approach which led to control of her malignant hypertension.

## Case presentation

**Fig. 1:**
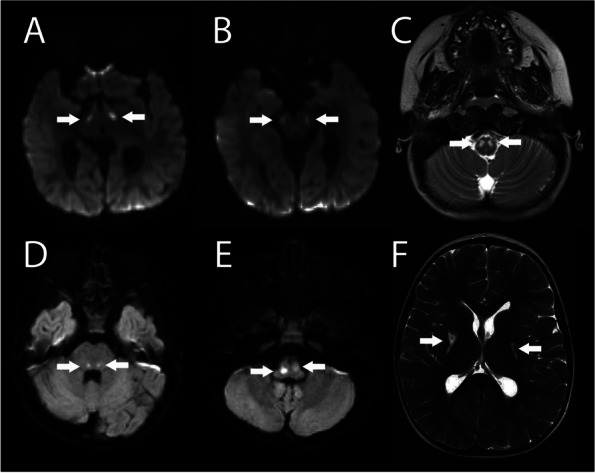
Images from MRI performed at 15 month (**A**-**C**) and at 46 month of age (**D**-**F**). The white arrows point to the key findings in each case. **A**-**B** are diffusion weighed images (DWI) representing acute infarcts in the sub-thalamic nuclei and brainstem. **C** is a T2-weighted (T2w) image demonstrating similar changes in the medulla and upper cord. **D**-**E** are DWI representing acute infarcts in the posterior pons and medulla. The findings are asymmetric in (**E**). **F** is a T2w image demonstrating the chronic infarcts in the basal ganglia

The patient was born at term to a 29-year-old Caucasian mother after uncomplicated pregnancy and delivery. At 6 months of age, she was evaluated for noisy breathing with a reassuring echocardiogram, chest radiograph, and nasal endoscopy. She then presented at 15 months of age for ongoing developmental motor delay, hypotonia, and failure to thrive. Labs at that time were notable for elevated lactate at 4.6 mmol/L (normal: 0.7–2.0 mmol/L), low bicarbonate of 13 mmol/L (normal: 20–30 mmol/L), elevated anion gap to 19 (normal: 2–15 mmol/L), and elevated pyruvate to 0.21 mmol/L (normal: 0.03–0.10 mmol/L). Initial ammonia was elevated at 66 mcmol/L (normal: 5–50 mcmol/L) but normalized on repeat labs. Magnetic resonance imaging (MRI) of the brain revealed multiple foci of bilateral and symmetric diffusion restriction in the sub-thalamic nuclei, brainstem, and upper cervical spine (Fig. 1A-C). Additional laboratory studies revealed elevated CSF lactate of 7.5 mmol/L (normal: 1.1–2.3 mmol/L), and pyruvic acid of 0.31 mmol/L (normal 0.03–0.11 mmol/L) with lactate/pyruvate ratio of 24 (normal: 10–30). She was started on a mitochondrial cocktail (coenzyme Q10, biotin, riboflavin, thiamine, alpha lipoic acid, and creatinine) along with sodium bicarbonate. A combined mitochondrial genome and mitochondrial nuclear gene panel revealed a pathogenic variant in the *MT-ND5* gene, designated as m.13513G > A;p.Asp393Asn with heteroplasmy of 69%, confirming the diagnosis of Leigh syndrome.

Her overall clinical course had been notable for Wolf-Parkinson White (WPW) syndrome, obstructive sleep apnea, global developmental delay, hypogammaglobulinemia, strabismus, and proximal renal tubular acidosis (RTA), for which she established follow up with nephrology. Her renal ultrasound was unremarkable except for two 2 mm cortical cysts in the right kidney. She was started on bicarbonate supplementation, initially sodium bicarbonate, which was later switched to Polycitra, Cytra K™. Her renal involvement had been limited to proximal RTA and mild elevation in urine protein/creatinine ratio until her presentation with malignant hypertension at 3 years of age.

The proband presented to the Emergency Department with tachycardia in the 170 s BPM for 3 days associated with insomnia and irritability. She was found to be hypertensive to 170 s/120 s mmHg and was admitted to the pediatric intensive care unit (PICU) for hypertensive emergency. She was initially treated with as needed intravenous labetalol and responded well. Workup was notable for a urinalysis with a urine protein/creatinine ratio of 1.2 mg protein/mg creatinine and 1+ glucose attributed to proximal tubular dysfunction. Repeat renal ultrasound revealed findings of increased echogenicity of the kidneys bilaterally. Serum sodium ranged 130–138 mmol/L potassium 3.9–4.1 mmol/L, chloride 95–100 mmol/L, CO2 23–31 mmol/L, BUN 22–23 mg/dL, and creatinine 0.16–0.22 mg/dL on day before and after urine studies and renal ultrasound were obtained. In the setting of stable serum creatinine and stable serum electrolytes, ultrasound findings were thought to be non-specific, and not indicative of acute kidney injury.

She was started on clonidine 2 mcg/kg q 8 hours with improvement in her hypertension but remained tachycardic and ultimately required the addition of low-dose oral labetalol. Given the severity of her hypertension, further workup included serum thyroid stimulating hormone, free T4, renin, aldosterone, and spot plasma metanephrines (Table 1). At the time plasma metanephrines were obtained patient was stable with BP ranging 118–122/50–83 mmHg and heart rate 110–140 bpm. Labetalol was stopped on admission given concern for pheochromocytoma and potentially causing the elevation via beta-blockade causing unopposed alpha-adrenergic activity. A computed tomography scan of her abdomen and pelvis with contrast did not show any concerning masses.

**Table 1:** Laboratory Values on Hospital Admission 1 Days 1 and 4 and Hospital Admission 2

Laboratory Test	Admission 1: Day 1	Admission 1: Day 4	Admission 2	Normal value
Thyroid Stimulating Hormone	0.86 mIU/mL	0.64 mIU/mL	NA	0.3–0.42 miU/mL
Free T4	1.35 ng/dL	NA	1.32 ng/dL	0.9–1.7 ng/dL
Plasma Renin activity	2.1 ng/mL/H	NA	1.1 ng/mL/H	1.5–3.5 ng/mL/H
Plasma Aldosterone	10 ng/dL	NA	210 ng/dL	< 40 ng/dL
Plasma Metanephrines	0.61 nmol/L	0.52 nmol/L	3.1 nmol/L	< 0.50 nmol/L
Plasma Normetaneprhines	2.6 nmol/L	2.7 nmol/L	10	< 0.9 nmol/L
Urine 24 Hr Metanephrine	n/a	102 mcg	NA	18–144 mcg/24H
Urine 24 Hr Normetanephrine	n/a	445 mcg	NA	29–145 mcg/24H
Urine Total metanephrines	n/a	547 mcg	NA	57–210 mcg/24H

*n/a* Data not available

She was discharged home after 11 days in the PICU on clonidine 14 mcg/kg/day, and doxazosin 0.06 mg/kg/day. After her discharge, she underwent MRI of chest, abdomen, and pelvis which did not reveal any masses concerning for malignancy or pheochromocytoma and was therefore restarted on labetalol 1.2 mg/kg/day to better control her blood pressure. After initiation of beta blocker and subsequent titration at home, she had an acute decompensation with altered mental status and was admitted second time to the PICU. Her exam was again notable for tachycardia (150 s -160 s) and malignant hypertension (BP 166/136), defined as systemic blood pressure greater than the 95th percentile plus 12 for age and height or greater than 140/90 with symptoms of end organ damage. Serum electrolytes during her second admission were as follows: sodium 140 mmol/L, potassium 5.2 mmol/L, chloride 103 mmol/L, CO2 23 mmol/L, BUN 25 mg/dL, and creatinine 0.23 mg/dL. Electrolytes remained stable and BUN and creatinine had normalized to 16 mg/dL and 0.18 mg/dL by day two of admission respectively. A repeat echocardiogram showed new mild dilation of her left ventricle and mild mitral regurgitation with slightly decreased left ventricular systolic function. Repeat serum metanephrines and normetanephrines were again elevated, higher than her first presentation (Table 1). This potentially explained her decompensation and worsening hypertension. Given her acute presentation and worsening clinical status, a repeat brain MRI revealed development of new acute infarcts in the posterior pons and medulla, in addition to chronic infarcts in the basal ganglia, both consistent with progression of Leigh syndrome (Fig. 1D-F). She was managed with a continuous nicardipine infusion and transitioned to a regimen of oral amlodipine (0.48 mg/kg/day), clonidine (14 mcg/kg/day), and atenolol (1 mg/kg/day) by hospital discharge. On this regimen, her hypertension has been well controlled below her 90th%ile for height and age.

## Discussion and conclusions

We report a 3-year-old female with Leigh syndrome caused by pathogenic variant in the *MT-ND5* gene who presented with malignant hypertension associated with elevated urine and plasma metanephrines. Subsequent evaluation failed to reveal a catecholamine-secreting tumor and a repeat brain MRI showed deterioration in her brainstem findings consistent with progression of her underlying mitochondrial disorder. This finding supported the diagnosis of dysautonomia as the cause for her severe hypertension. Given that her plasma aldosterone concentration was 210 ng/dL during her second admission, a diagnosis of primary aldosteronism can also be considered. Although no confirmatory testing with adrenal vein sampling tests were obtained, we though this less likely given MRI abdomen and pelvis with and without contrast showed normal adrenal glands without evidence of adrenal tumors or hyperplasia. In addition, her plasma aldosterone/renin ratio was not suggestive of hyperaldosteronism in the setting of elevated catecholamines.

Malignant hypertension is a reported, but under-recognized symptom of mitochondrial disease [3]. The etiology of hypertension in Leigh Syndrome is also poorly understood and can even be transient [4]. Elevated catecholamines have been reported, but are not a common feature in patients with Leigh Syndrome. There is one previously described case report of a child with Leigh syndrome caused by the *MT-ND5* m.13513A > G mutation presenting with malignant hypertension [3]. The patient presented with renal salt wasting, proximal tubular dysfunction, and syndrome of inappropriate antidiuretic hormone in association with rapidly progressive hypertrophic cardiomyopathy, and WPW-like conduction defect. That child required electrical cardioversion and had systemic hypertension with associated elevation of serum and spot urine catecholamines similar to the patient described in this report. The elevation in plasma and urine metanephrines in our index patient, along with her acute presentation of hypertensive emergency and diaphoresis, was likely a centrally mediated process secondary to her mitochondrial disorder and concomitant cellular dysfunction and/or frank cell death. There were two additional reports association acute severe hypertension with Leigh syndrome but no molecular data provided [5, 6]. In addition, a fatal hypertensive crisis as presentation of mitochondrial complex I deficiency has also been described in one patient [5].

The *MT-ND5* gene codes for subunit 5 of the mitochondrial complex I (NASH:ubiquinone oxidoreductase), which accepts electron from NADH, mediates their transfer to Coenzyme Q10, and participates in pumping protons into the mitochondrial intermembrane space. The proton gradient produced is utilized through oxidative phosphorylation to generate ATP, the ubiquitous intracellular energy source for all cells. Organs with high energy demands are therefore most often directly affected in diseases of oxidative phosphorylation. These organs include the heart, brain, and skeletal muscle but can affect any organ.

The control of central autonomic activity to the cardiovascular system occurs mainly in the brain stem region [7]. This part of the brain plays a pivotal role in control of sympathetic activity with direct effects on systemic blood pressure. Specifically, the rostral ventrolateral medulla oblongata appears to be involved in neurogenic hypertension. The patient described in this report had new areas of infarction in her medulla and other regions of the brainstem likely causing her hypertension.

We suspect her continued labile blood pressures were due to centrally mediated autonomic dysfunction and were an indicator of disease progression. Her subsequent MRI findings supported disease progression with new diffusion restriction involving bilateral posterior tracts in the posterior pons and medulla, along with chronic bilateral infarcts in the basal ganglia (Fig. 1D-F). Prior case reports of patients with Leigh syndrome with brainstem, specifically nucleus tractus solitarius, involvement, had also presented with significant deterioration in neurological function [8]. Her antihypertensive regimen was chosen to target autonomic dysfunction. This included alpha action (clonidine), beta blockade (atenolol) and calcium channel blockade with amlodipine. She had been started on doxazosin for alpha blockade but this was thought less effective given her presentation with malignant hypertension while on that medication.

The prognosis of patients with Leigh Syndrome remains poor with most succumbing to respiratory and or cardiac failure in the first 1–2 years of life. As such, respiratory compromise and heart failure are common manifestations. In addition, typical symptoms of autonomic dysfunction with hypotension, flushing and tachycardia are also common. With treatment of her hypertensive symptoms, our patient has continued to survive with reasonable quality of life beyond this time. This report highlights the need to recognize possible autonomic dysfunction in mitochondrial disease and illustrates the challenges for accurate and prompt diagnosis and subsequent management of the associated manifestations. Hopefully with better recognition of this potential, we can begin to improve the prognosis for these patients.

## Data Availability

There was no data associated with this case report.
